# A Citizen Science Trial to Assess Perception of Wild Penguin Welfare

**DOI:** 10.3389/fvets.2021.698685

**Published:** 2021-07-27

**Authors:** Rafael Freire, Melanie Massaro, Simon McDonald, Philip Trathan, Christine J. Nicol

**Affiliations:** ^1^Institute for Land, Water and Society, School of Agricultural, Environmental and Veterinary Sciences, Charles Sturt University, Albury, NSW, Australia; ^2^Spatial Data Analysis Unit, Charles Sturt University, Albury, NSW, Australia; ^3^British Antarctic Survey, Cambridge, United Kingdom; ^4^Royal Veterinary College, University of London, London, United Kingdom

**Keywords:** animal welfare, Spheniscidae, anthropogenic impact, five domains model, welfare assessment

## Abstract

Wild penguins are facing increased threats to their populations and their welfare as a consequence of human activities. Understanding the perception of animal welfare is essential to identify ethical concerns related to the negative impact of anthropogenic factors on wild species and to guide conservation efforts that reflect societal values. Since penguin conservation is of general interest, we examined the human dimension of welfare assessment across a range of interest groups concerned with penguins, seabird biology and wildlife conservation. We provided participants with a Penguin Welfare Assessment Tool (PWAT) based on the five domains model. The PWAT supports consideration of the impact of four physical aspects on welfare-relevant mental states. Bibliometric analysis of keywords from 347 scientific articles indicated that penguins around the world face five main types (themes) of anthropogenic factors and we then developed five hypothetical scenarios, each related to one theme. Seventy-five participants scored the overall impact of the events described in the scenarios on penguin welfare as negative using the PWAT. Participants rated short-duration, high-intensity events (i.e., being trapped in a ghost fishing net) as having a significantly more severe impact on penguin welfare than low-intensity, long-duration events (*P* < 0.0001). Scores provided by participants for each domain for each scenario were largely as expected and we found good correlation (all *P* < 0.0001) between the physical domains and “mental state” for all scenarios, indicating that the tool was facilitating the participants' assessment of welfare. No evidence was found that experience of working or studying penguins, or indeed any other demographic factor investigated, influenced the assessments of welfare. We found little agreement between participants in the scores provided (unalike scores mostly between 0.7 and 0.8), and agreement between participants with experience of working with penguins was no better than between participants without such experience. We discuss the possibility that low agreement within different interest groups may be improved by providing more scientific information to support the evaluation of penguin welfare. We conclude that scientific knowledge of penguin biological responses to anthropogenic factors is vital to support the evaluation of wild penguin welfare by the public and other stakeholders.

## Introduction

Although concern for animal welfare has been mainly directed toward animals with which we have close contact, increasing attention is paid to the welfare of wild animals, at least by the public (1, 2) and conservation scientists (3–9). However, a crucial foundation to support constructive collaboration between various scientific disciplines, interest groups and other stakeholders is to understand how different groups perceive wild animal welfare. Perception of animal welfare depends on people's values, experiences, convictions and factual knowledge (10, 11). Understanding variation in people's perception of animal welfare is a vital component in operationalizing animal welfare actions, as it can identify wider ethical concerns and guide the approach taken to increase broader awareness of animal welfare (12). It can also have practical applications, such as in ranking areas for priority action (13).

One barrier to wider collaboration on wild animal welfare matters is that people may perceive the impact of negative events on the welfare of wild animals differently. Due to the complexity of evaluating the welfare of wild animals, welfare assessment has up to now been undertaken by the authors themselves [e.g., (14)] or by a panel with expertise in animal welfare [e.g., (15, 16)]. To scientists, animal welfare concerns the state of an individual animal with regards to its attempt to cope with its environment (17) and welfare assessment typically evaluates the impact of physical and mental aspects on the animal's state. Little is known about how interest groups perceive and evaluate animal welfare, though in the context of farm animals, notions about the animal's natural environment appear to be an important consideration (18). Engaging diverse participants in scientific endeavors (termed “citizen science”) provides an explicit structure to elicit opinion about animal wefare which can benefit scientific and community goals (19). Additionally, a citizen science framework has already been proposed to progress public understanding of science and support a multi-way dialogue of engagement with beneficial environmental outcomes (20, 21).

Within animal welfare science, the five domains model (FDM) has emerged as a valuable tool for the systematic and structured assessment of animal welfare (22). The FDM considers the effect of four physical domains (nutrition, environment, health, and behavior) on a fifth, subjective experience state called an “affective,” or “mental,” state. The advantage of the FDM is that it allows the identification of a wide range of impacts, produces a relative ranking that is easy to interpret, highlights knowledge gaps and can be modified as new information becomes available (23). Although the FDM was not designed specifically for use within a citizen science framework, its simplicity of use lends itself to this goal. Here, we examined the perception of wild penguin welfare across a range of groups with interests in penguins, seabird biology and wildlife conservation through their use of a welfare assessment tool based on the FDM.

We had several reasons for choosing penguins (*Spheniscidae*) to assess interest groups' perception of wild animal welfare. First, although the distribution of penguins is restricted to the southern hemisphere, members of this family can be found along the coasts of all continents and large islands of the southern hemisphere (Africa, South America, Oceania, and Antarctica), providing a potentially diverse sample of participants. Second, penguins are one of the most well-known groups of birds across the globe, due to numerous wildlife documentaries and articles in the media. For example, on Wikipedia, the “Emperor Penguin” page receives one of the highest views of any bird (24). Familiarity with a species is essential to assess welfare, hence it was important to choose a group of birds that is relatively well-known and recognizable to most people around the globe. Third, penguin conservation is a major challenge and penguins are considered sentinels of the marine environment providing valuable conservation information on the southern ocean environment (25). Ten of the 18 species of penguins are considered endangered or threatened by the IUCN (26), and many are impacted by a range of human-induced environmental changes (hereafter termed anthropogenic factors) (27).

This study aimed to assess the perception of wild penguin welfare of participants who have an interest in penguins, seabird biology and wildlife conservation. We first developed a Penguin Welfare Assessment Tool (PWAT) based on previous tools that assess the welfare of wild animals using the five domains model [e.g., (16, 28)] but we adjusted the tool to take into account penguin habitat and biology. To identify anthropogenic factors with the potential to impact penguin welfare, we undertook a search of the recent literature (2010–2020) and grouped keywords from these articles using the VOSviewer bibliometric and network analysis software (29). We then developed five hypothetical scenarios based on the anthropogenic factors identified by our network analysis describing the effect of five events on penguin biology. The scenarios were distributed to interest groups that were asked to provide their perceived assessment of the impact of anthropogenic factors on penguin welfare using the PWAT. We hypothesized that those participants with experience of working or studying penguins would have a higher level of intra-group agreement than those without working experience of penguins. We examined “agreement” by calculating the coefficient of unalikeability (30, 31). We also investigated the level of agreement among different groups of participants and collected a range of demographic factors in order to further explore potential variation in welfare assessments.

## Method

### Development of the PWAT Tool

**Figure 1:**
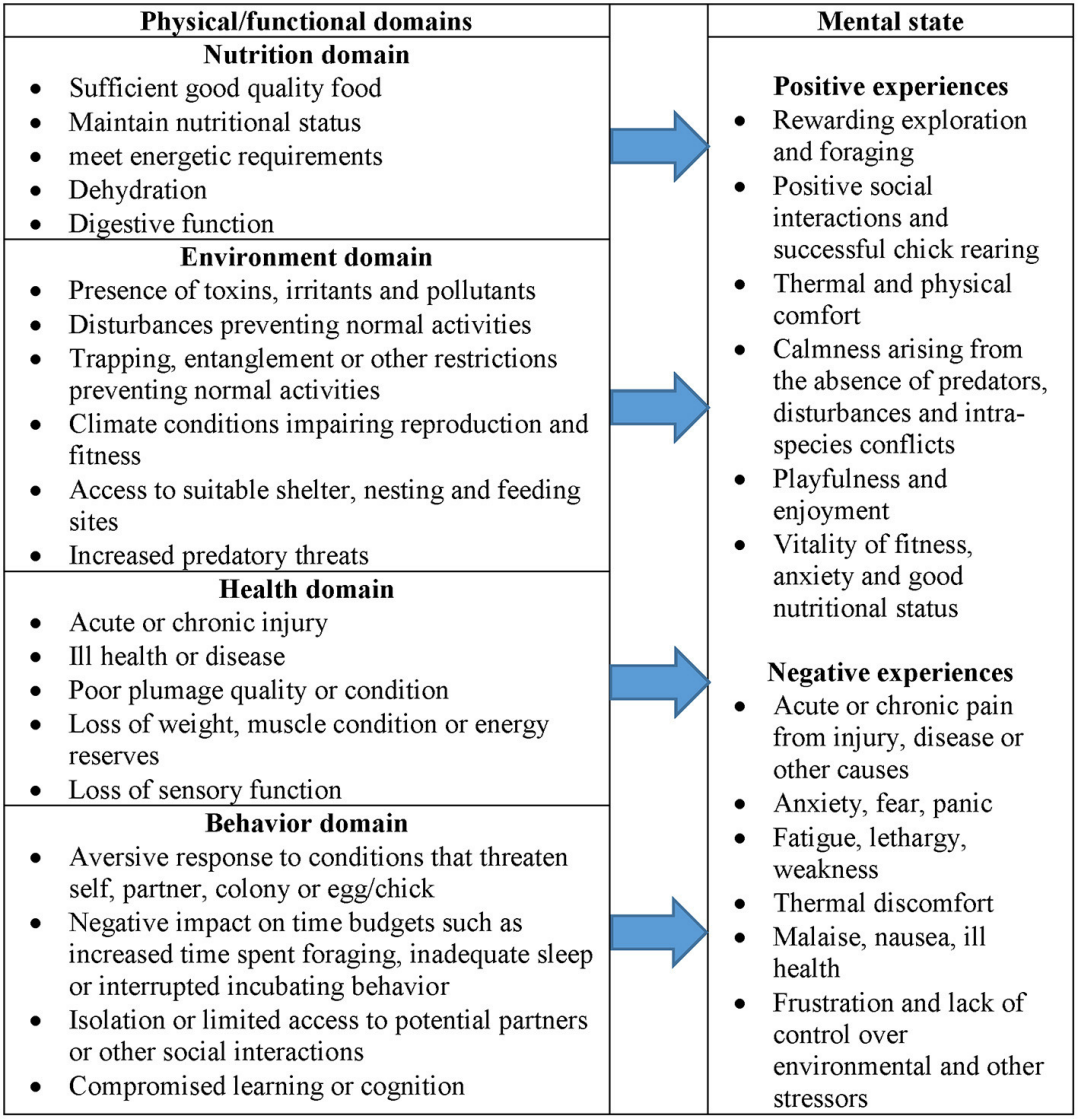
Penguin welfare assessment tool.

We developed a penguin welfare assessment tool (Figure 1) based on similar tools that were built for whales and other cetaceans (16) and for wild horses (28). The tool requires users to assess the impact of four physical domains (nutrition, environment, health and behavior) and mental state on penguin welfare. Although participants were not asked to assess “positive” mental states, we included positive experiences in the model to assist assessors that may have been unfamiliar with the concept of “mental state” with respect to animals.

### Selection of Scenarios of Anthropogenic Effects on Penguins

We wrote five scenarios that described the effect of five anthropogenic-driven events on penguins, taking care to restrict our description to effects on measurable biological effects. To choose the scenarios we undertook various exploratory literature searches to identify the subject of each scenario. Our preliminary searches indicated that a broad search strategy with subsequent by-hand filtering would avoid excluding relevant literature. We also decided that the search should focus on relatively “newer” literature to ensure that the anthropogenic effects were currently relevant. An ISI Web of Science search was used on the 29/6/2020 for the period 2010–2020. Search terms were “penguin or Spheniscidae” (the plural “penguins” did not yield any more articles). Indexes used were SCI-expanded, SSCI, A&HCI, CPI-SSH, ESCI, CCR-expanded and IC, and all languages and all document types were included. By-hand filtering involved selecting records that involved possible anthropogenic effects. The approach was to be inclusive of any item that could potentially involve “anthropogenic effects,” so for example most papers on the impact of “climate” or “disease” were included even though some of these impacts may not have been caused by humans. A csv file of included and excluded full references is available from the corresponding author.

A network of the keywords from the resulting dataset comprising of 347 citations on anthropogenic effects was created using VOSviewer (Supplementary Figure 1) (29) which the software grouped into five clusters (themes). The most common keywords in each cluster were used to create five scenarios that described the impact of the events on the biology of the penguins (Table 1). The five scenarios varied in the length of time that penguins experienced the events, from hours (ghost fishing net; lost fishing gear is often referred to as “ghost gear,” as it continues to entrap animals) to years (mercury accumulation), and also in the intensity of the events. The five scenarios written using common words from each cluster are shown in Box 1.

**Table 1:** Most common words in the five clusters of articles on anthropogenic effects on penguins identified by VOSviewer, and titles of the five scenarios.

**Cluster**	**Human disturbance (Yellow)**	**Climate change (Blue)**	**Pollution (Red)**	**Fisheries (Purple)**	**Health (Green)**
Common words	Behavior Stress Tourism Responses Magellanic Breeding success	Population dynamics Adelie Southern ocean Competition Reproductive success	Mercury Antarctica Feather Food-web Accumulation	Conservation Magellanicus Impacts Patagonia	Mortality African penguins Demersus Prevalence Rehabilitation
Title of scenario	Effect of tourism on nesting female Magellanic penguin	Effect of increased ice melt at nesting sites on male Adélie penguin	Effect of mercury accumulation on mature Chinstrap penguin	Yellow-eyed penguin caught in a ghost fishing net	IBDV^*^ infection in young African penguin
Abbreviated title of scenario	Tourism	Ice melt	Mercury accumulation	Ghost fishing net	IBDV infection

*Network is shown in the Supplementary Material*.

* *Infectious bursal disease virus*.

Box 1 **Five hypothetical scenarios based on the key words of the five clusters identified by VOSviewer software.**

Effect of tourism on female Magellanic penguin

A 4 year old female Magellanic penguin has recently begun breeding and is part of a large colony in South America that is open for tourism. Tourism has been popular at this site since 2001 and penguins are subjected to human visits for most of the day during most of the breeding season (September to February). This female's nest is in a burrow that has an entrance under a small bush located near a retractable rope separating the visitors' walkway from the nesting birds. She incubated two eggs and successfully reared one chick this year, and this chick appeared to be of average size and weight for its age. The chick had a higher corticosterone increase in response to capture and restraint compared to chicks from a nearby site that is not visited by tourists, though the difference in stress response between chicks from the two sites was similar near the time of fledging. The second egg did not hatch in the 1st year, which is not unusual for this species in either tourist-visited or undisturbed sites.

On this day, this female is rearing one chick which is of average size and weight for her age having incubated two eggs. She shows an elevated heart rate response when approached by visitors and her basal blood corticosterone levels are higher than neighboring penguins that have been nesting at this site for more than 5 years. When she moved off the nest in the afternoon, she stared at a group of visitors and gave an alarm call before showing aggressive behavior toward them.

Effect of increased ice melt at nesting sites on male Adélie penguin

In spring, a 14 year old male Adélie penguin was nesting on a west-facing narrow pebbly beach, which has a glacier that extends along the entire the eastern side of this beach. This penguin had successfully reared chicks at this site in previous seasons. This year, however, the warmer than average temperatures and unusual northerly winds were melting the glacier ice more than in previous years.

On this day, the penguin had just returned from a feeding trip. Silverfish (*Pleuragramma antarcticum*), which is one of the penguin's main prey, is not as abundant as usual and this last foraging trip was 10% longer than in previous years. Water run-off surrounded this penguin's nest as he approached it, and in the afternoon water entered the nest. The penguin was observed to shiver for 20% more of the time this day, and his body weight was 5% lower, compared to the same time the previous year. His feather condition was also poor and the feathers on his belly were waterlogged. Basal corticosterone levels were no different this day to previous years.

Effect of mercury accumulation on mature Chinstrap penguin

On this day in April, a 14 year female Chinstrap penguin is moving north toward winter feeding grounds in the South Atlantic ocean. Her body weight is 5% lower than average for penguins her age, and she has a depressed immune system. Mercury concentration in her feathers (5 μg.g^−1^) and blood (1.5 μg.g^−1^) are higher than usual for penguins her age. She has elevated baseline plasma corticosterone levels and significantly decreased levels of luteinising hormone compared to penguins with lower feather and blood mercury levels. While feeding, this penguin dived to an average depth of around 22 m with an average duration of around 50 s, which is about 15% shallower and 20% shorter than average for Chinstrap penguins foraging at the same locations.

Before this day, this penguin had always nested on an island near the north end of the Antarctic peninsula. The soil of the island where this penguin nested has around 30 ng.g^−1^ of mercury, and local environmental conditions have been found to enhance the production of derivatives of mercury.

Yellow-eyed penguin caught in a ghost net

On this day, a 7 year old female Yellow-eyed penguin in good health was feeding in the waters south of the New Zealand shelf when she was caught in a piece of discarded gillnet that had been floating on the surface for some time. Although she could still raise her head to breath, she struggled violently in an attempt to free herself from the net. During her struggles, she received lacerations to the edges of her flippers and feet. The net was also around her neck, and as she struggled she developed a 7 cm long cut that was in places up to 7 mm deep. The penguin also suffered subcutaneous bruising around her neck with some internal bleeding in the thoracic and abdominal cavity. Her feathers over the head and neck region are water-logged. Three hours after being entangled, a combination of her struggling and pecking at the net released her from the net, and she was able to swim clear of the debris.

IBDV infection in young African penguin

On this day, a 5 months old male African penguin was foraging off the coast of South Africa. This penguin had lived in this area since fledging in August. The penguin was healthy in his earlier years, but unusually for penguins he is infected with infectious bursal disease virus (IBDV). His Bursa of Fabricus is swollen and the membranes of his trachea are thickened and are producing large amounts of mucus. This penguin is currently immunosuppressed and weighs 10% less than average penguins of the same sex and age. His basal corticosterone levels are unknown. He is visibly more lethargic and weaker than other penguins. IBDV is a pathogen of domestic chickens and does not usually infect or cause clinical signs in penguins. However, this penguin may have contracted the virus by coming into contact with feral domestic chickens on the shores of the South Africa mainland.

### Survey Method

A survey was made available to participants with a likely broad interest in penguins, birds and/or wildlife conservation to investigate how they would use the PWAT. A link to the Survey was advertised to members of the Australasian Seabird group, Seabird.net, Global Penguin Society, IUCN Penguin Specialist Group, Pew Charitable Trust, Birdlife, Penguin Foundation and Penguin World in October 2020. The survey was kept open for 6 weeks with two reminders sent at 2 and 4 weeks after opening.

Participants were first asked to familiarize themselves with the PWAT which was described as a tool to guide the systematic assessment of welfare by first ensuring a broad consideration of physical and functional states associated with each scenario. The PWAT then requires participants to make a judgement about the associated subjective mental state that a sentient animal may experience. Each scenario was then presented in random order *via* SurveyMonkey, and the participants were asked to rate the maximum impact of the event on the physical domains (nutrition, environment, health, and behavior) using a Likert scale ranging from “no harmful impact” to “severe harmful impact.” Participants were next asked to rate the overall effect of the event on the penguin's mental state using a Likert scale ranging from “no negative effect” to “severe negative effect.” Participants were also asked how long they thought the given mental state would persist following the event (six point scale; 1–6 days, 1–4 weeks, 1–6 months, 7–11 months, 1–2 years, and more than 2 years).

In order to obtain an indication of the level of personal experience participants had with penguins, we asked how long they had worked or studied penguins. We also asked participants to state their main area of scientific or work interest with the following options; penguin biology or conservation, other biology or conservation, ecology, wild animal management or other. Finally, in order to obtain a clearer understanding of the profile of our participants we asked them their age bracket (six point scale), their usual place of residence, and if they belonged to a conservation, animal welfare or environmental organization. Participants were also asked their dietary habits (e.g., omnivore, vegetarian) because concern for animal welfare has been shown to be a primary driver for people adopting restricted diets (32). A copy of the survey is available from the authors upon request. The study was approved by CSU's Human Research Ethics Committee (H20292).

### Statistical Analysis

Seventy five respondents provided scores for at least one scenario. We first ran a linear model (33) to assess whether there were any effects relating to the order (first, second, etc.) in which participants had received each scenario, using the score given to mental state as the output variable. We calculated the slope for each participant. Only five (out of 75) participants showed a significant slope when plotted against order (two positive slopes and three negative slopes). We concluded that there was no evidence that the order of presentation of the scenarios influenced the scores given so dropped the order of presentation of each scenario from all subsequent analyses.

We next considered whether fitting multi-level linear mixed effects models to our ordinal outcome data from our Likert scale would be appropriate. The Linear analyses described below were repeated by performing the same modeling using Penalized Quasi-Likelihood (PQL) and Markov Chain Monte Carlo (MCMC) methods, both of which have been suggested to be superior to linear modeling for analyzing ordinal data in some circumstances (34). The results of the PQL and MCMC analyses (33) did not provide any different outcomes in terms of significant and non-significant effects to the Linear analysis. We therefore opted to only present the results of the Linear models.

Since scores for the “mental state” domain represents participants' overall assessment of welfare, we first analyzed response scores given by participants for mental state using a General Linear Mixed Model with scenario as a factor, and participant identity as the random factor (33). Spearman rank order correlations were undertaken to examine the relationship between physical domain scores and the score given for the mental state domain.

The questions of area of research or work expertise and experience of working with penguins were answered by 64 participants. A summary of the number of participants in each category is provided in Supplementary Table 1. We reclassified responses to the experience question into four categories because there were too few participants in the <1 year (*N* = 2), 1–2 years (*N* = 3), 3–5 years (*N* = 8), and more than 20 years (*N* = 4) categories. The final categories were therefore no experience of working with penguins (*N* = 22), <5 years' experience (*N* = 13), 6–10 years' experience (*n* = 17) and more than 10 years' experience (*N* = 12). A General Linear Mixed Model (33) was used to compare response scores on the five domains by expertise (four levels) and length of experience of working or studying penguins (four levels), with participant identity as a random factor.

To examine the influence of demographic factors on response scores we fitted General Linear Mixed Models using R (33) based on all possible combinations of the following predictors: age, organization, region, dietary habits, expertise, and experience. Participant identity was included as a random factor. Models were ranked using an information theoretic approach based on Akaike's Information Criterion (35).

In our initial investigation into the level of agreement between participants it became apparent that the increments of the original scale were too fine to allow a meaningful examination of agreement. We therefore recalculated our scale into a five point scale ranging from 1 (slight or no harmful impact) to 5 (major to extreme harmful impact). Agreement between participants with varying amounts of experience of working and studying penguins, and between participants with different areas of expertise was examined using the coefficient of unalikeability (31) which indicates how often observations differ from one another. The coefficient of variability (*u*_2_) represents the proportion of possible comparisons (pairings) which are unalike and ranges from 0 to 1, with the higher the value, the more unalike the data are.

## Results

### Impact of the Events Described in the Scenarios on Penguin Welfare

**Figure 2:**
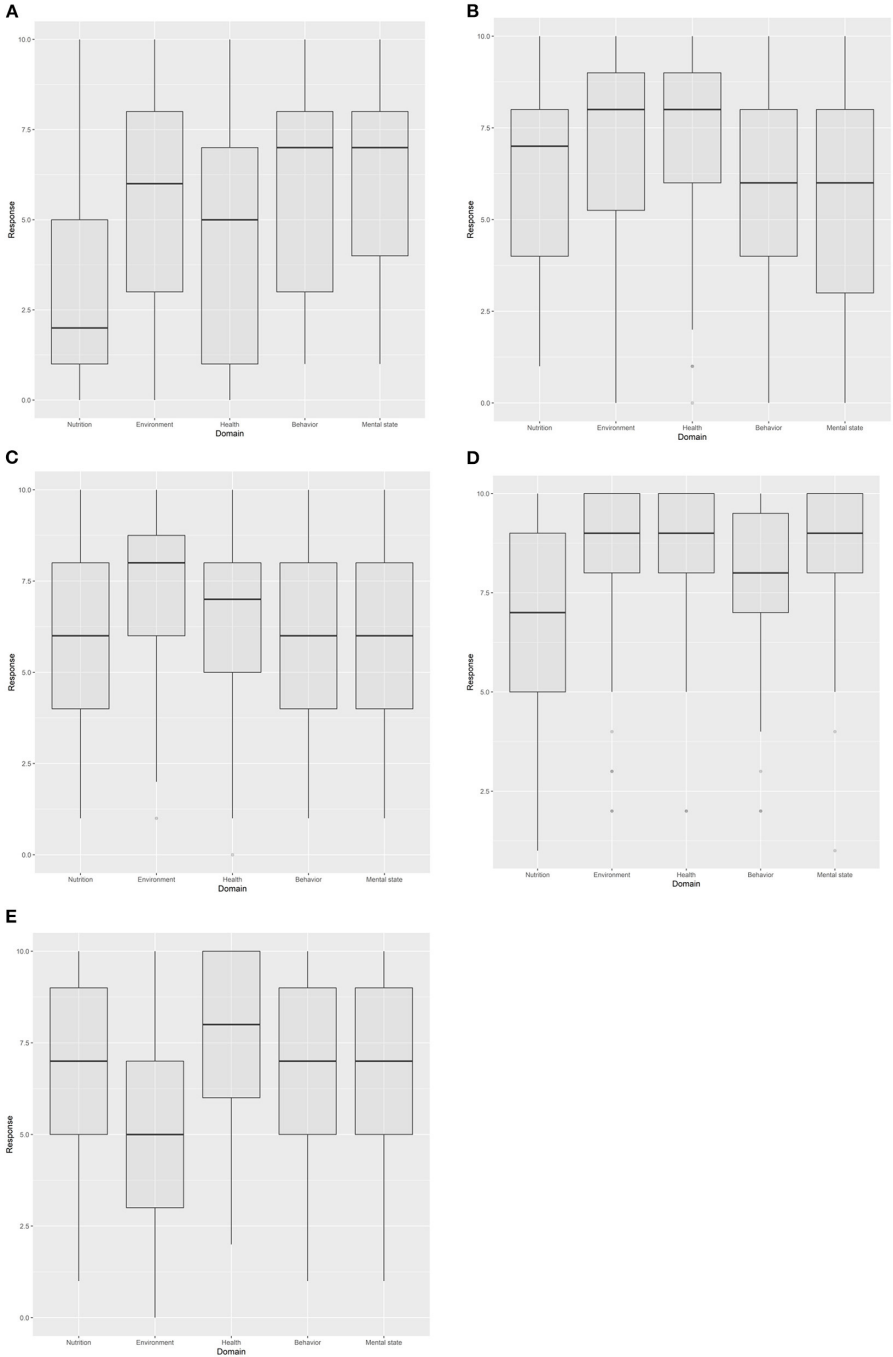
Summary of scores given by respondents for each of the five scenarios: **(A)** effect of tourism on female Magellanic penguin, **(B)** effect of increased ice melt at nesting sites on male Adélie penguin, **(C)** effect of mercury accumulation on mature Chinstrap penguin, **(D)** Yellow-eyed penguin caught in a ghost fishing net, and **(E)** IBDV infection in young African penguin.

**Figure 3:**
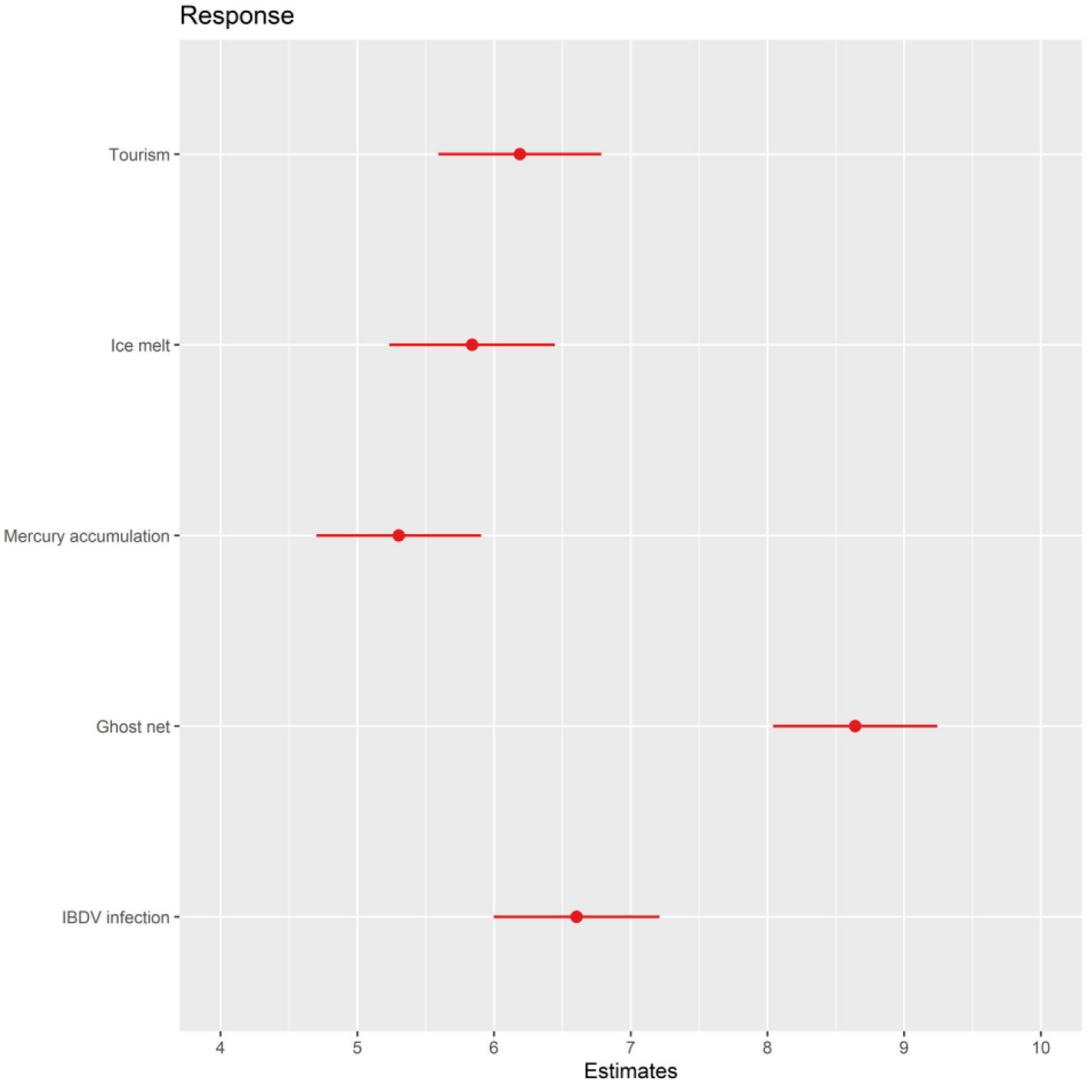
Parameter estimates and 95% Wald confidence intervals for the response scores given for mental state for each scenario.

Seventy-five participants provided response scores for at least one scenario. Results (Figures 2A–E) indicated that the perceived harmful impact of events on most domains was considerable for all penguins. Participants scored the “ghost fishing net” scenario as having the most negative effect on the penguin's mental state [GLM, *F*_(4, 259)_ = 36.6, *P* < 0.0001; Figure 3]. No significant difference in the extent of negative effect was found between the “tourism,” “ice melt,” and “mercury accumulation” scenarios (Figure 3).

**Figure 4:**
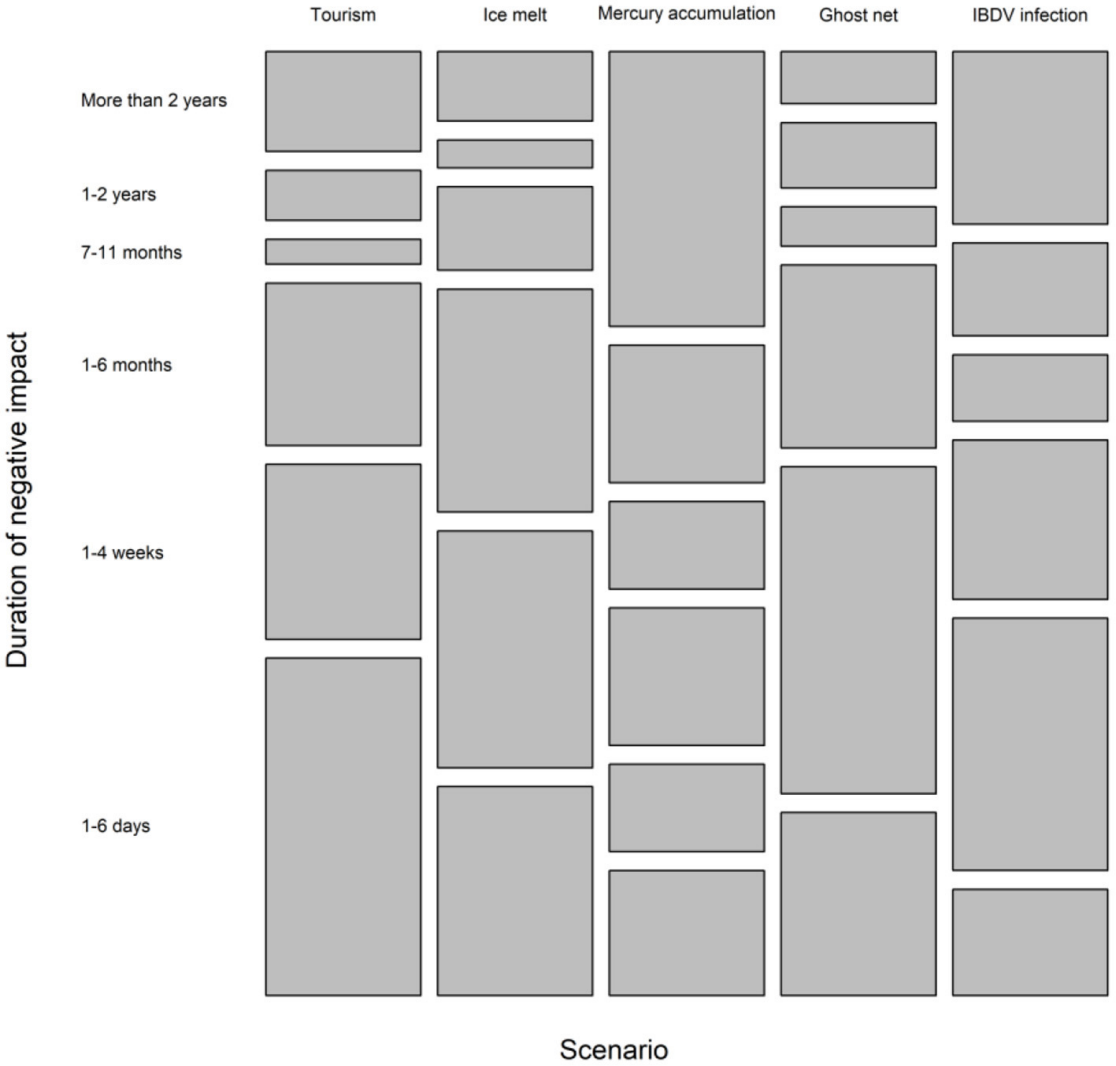
Percentage of responses given for the duration of negative impact for each scenario.

The majority of respondents (>70%) considered that the negative effect of tourism, ice melt and the ghost fishing net would last <6 months (Figure 4). The exception was mercury accumulation, with the highest percentage of respondents (32%) indicating that the effects lasted for more than 2 years (Figure 4). It was difficult to discern participant's overall view of the duration of negative effect of IBDV infection, as 42% or participants considered that the negative effects would last <4 weeks, and 31% that the negative effects would last more than 1 year.

### Correlation of Scores on the Physical Domains With Mental State

Results (Figures 2A–E) indicated, that in general, the scores for each domain reflected the likely main impact on the penguins. Assessment of “environment” and “behavior” were scored the highest for the scenario on tourism; “environment” scored the highest for the scenario on ice melt; “health” and “environment” scored the highest for both the scenario on mercury accumulation and being trapped in a ghost fishing net and “health” scored the highest for the scenario on IBDV infection. Significant correlation was found between the scores for the physical domains and the mental state domain (all *P* < 0.0001; Table 2). For four of the five scenarios, the score given for “behavior” was more closely correlated with the score given for “mental state” than scores for the other physical domains (Table 2). The exception was for the scenario on the effect of tourism, for which the score given for “environment” was more closely correlated to the “mental state” score than the scores for other physical domains. Nonetheless for the latter scenario, “behavior” was highly correlated with mental state (*r* = 0.819). It is also interesting to note that at least one physical domain scored higher than the mental state domain for the ice melt, mercury accumulation and IBDV infection scenarios (Figure 2).

**Table 2:** Spearman Rho coefficients of each of the four physical domains and the mental state domain for each of the five scenarios.

**Scenario**	**Nutrition domain**	**Environment domain**	**Health domain**	**Behavior domain**
	Correlation with score given for “mental state” (*r*)			
Tourism	0.445^*^	0.872^*^	0.672^*^	0.819^*^
Ice melt	0.635^*^	0.681^*^	0.666^*^	0.806^*^
Mercury accumulation	0.573^*^	0.407^*^	0.586^*^	0.686^*^
Ghost fishing net	0.483^*^	0.549^*^	0.554^*^	0.640^*^
IBDV infection	0.616^*^	0.587^*^	0.622^*^	0.829^*^

* *P < 0.0001*.

### Influence of Experience of Working With Penguins and Demographic Factors on Participants' Scores for Each Domain

**Figure 5:**
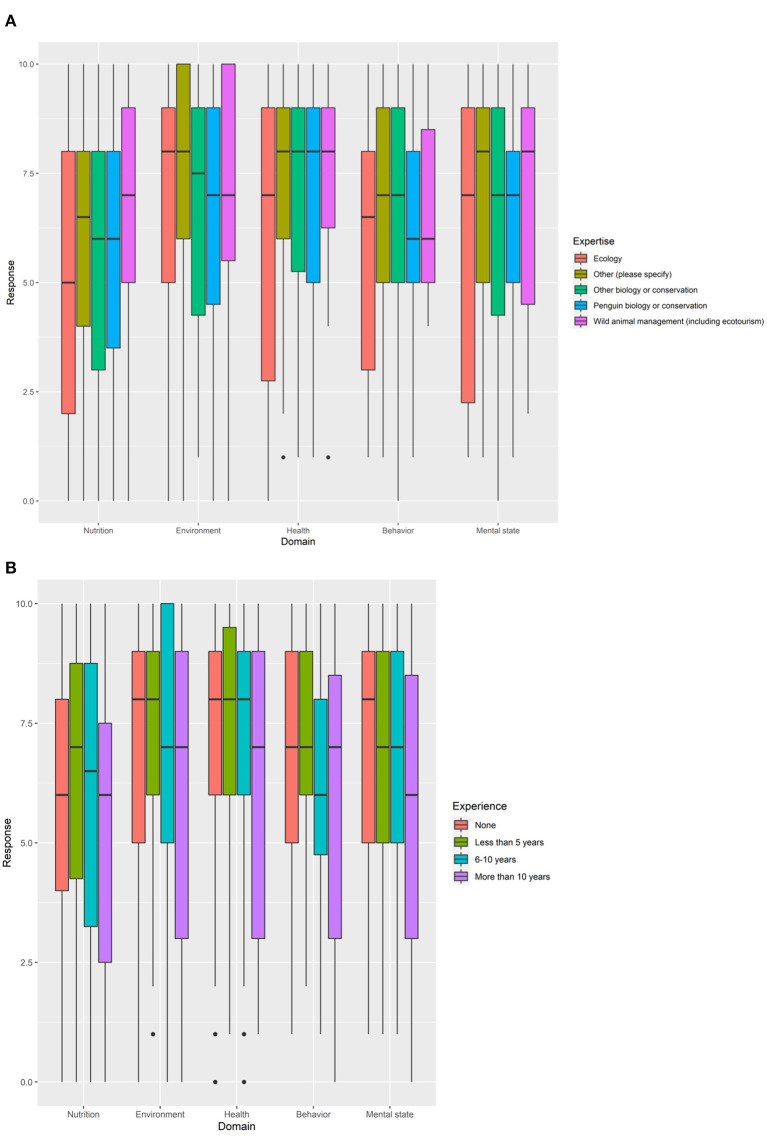
Summary of responses for each domain for participants with different **(A)** areas of scientific or work interests and **(B)** levels of experience of working or studying penguins.

Eighteen participants had experience of working with penguins in biology or conservation areas, with a further 22 participants having experience of working with penguins in other areas (Supplementary Table 1). No evidence was found that the main area of scientific expertise or work interest influenced the scores given for each domain [GLMM, *F*_(4, 59)_ = 0.82, *P* = 0.65]. On the whole, the “penguin biology or conservation” group of participants (*N* = 18) scored similarly to other groups (Figure 5A). For participants that had previously worked with penguins (*N* = 42), the length of time that participants had worked with or studied penguins had no effect on the scores given for each domain [GLMM, *F*_(3, 59)_ = 1.14, *P* = 0.34; Figure 5B].

We had no participants in the 18–24 age bracket, but otherwise the age distribution of participants was even (between 9 and 15 participants in the five brackets between 25 and 65+ years). Forty two (out of 63 responses to this question) participants belonged to organizations such as the RSPCA (*N* = 4), WWF (*N* = 6) and ornithology groups such as Birdlife (*N* = 14). Most participants were from the Australasia or pacific region (*N* = 39), with the next highest group from Europe (*N* = 14). Most participants indicated that they had an omnivorous diet (*N* = 47), with 14 participants indicating a vegetarian or vegan diet. Linear mixed modeling of all possible demographic factors on response scores indicated that none of our factors influenced response scores. As an indication of our findings, the top ranked model using AIC included organization [GLMM, *F*_(1, 56)_ = 1.4, *P* = 0.22] and dietary habits [GLMM, *F*_(1, 56)_ = 1.1, *P* = 0.37]. Outcomes of all models are available from the authors.

### Agreement Between Participants on the Extent of the Harmful Impact on the Penguins

On a 5-point scale, the majority of unalike values were in the 0.7–0.8 region, indicating that participants disagreed on the impact on the penguins on 70–80% of occasions (Table 3). The exception was the ghost fishing net scenario (Figure 2D), where more than half the unalike scores were below 0.5 indicating that participants showed better agreement on the impact of this event on the physical and mental state of the penguin when compared to the unalike scores for the other scenarios.

**Table 3:** Unlike scores based on the 5-point-scale for each domain and scenario for participants without experience of penguins (*n* = 22) and participants with prior experience of working with penguins.

**Domain**	**Scenario**	**No experience (*n* = 22)**	**Experience <5 years (*n* = 13)**	**6–10 years experience (*n* = 17)**	**More than 10 years experience (*n* = 12)**
Nutrition	Tourism	0.75	0.87	0.74	0.65
	Ice melt	0.79	0.79	0.79	0.63
	Mercury accumulation	0.69	0.72	0.76	0.78
	Ghost fishing net	0.77	0.78	0.78	0.72
	IBDV infection	0.73	0.73	0.74	0.80
Environment	Tourism	0.80	0.79	0.83	0.85
	Ice melt	0.75	0.65	0.72	0.74
	Mercury accumulation	0.74	0.76	0.78	0.76
	Ghost fishing net	0.52	0.52	0.35	0.62
	IBDV infection	0.77	0.82	0.73	0.86
Health	Tourism	0.81	0.82	0.67	0.73
	Ice melt	0.73	0.79	0.59	0.81
	Mercury accumulation	0.71	0.73	0.72	0.81
	Ghost fishing net	0.54	0.41	0.57	0.53
	IBDV infection	0.66	0.56	0.64	0.79
Behavior	Tourism	0.78	0.79	0.79	0.78
	Ice melt	0.75	0.76	0.70	0.73
	Mercury accumulation	0.75	0.71	0.66	0.80
	Ghost fishing net	0.69	0.62	0.58	0.68
	IBDV infection	0.69	0.76	0.64	0.83
Mental State	Tourism	0.77	0.77	0.81	0.83
	Ice melt	0.77	0.78	0.74	0.78
	Mercury accumulation	0.81	0.77	0.78	0.84
	Ghost fishing net	0.50	0.38	0.44	0.60
	IBDV infection	0.76	0.79	0.79	0.83

Examination of the unalike scores on the 5-point scale (Table 3) did not support the prediction that people with experience of working or studying penguins were more likely to agree on the extent of harmful impact of each event described in the scenarios on the penguins. Again, there was better agreement within all sub-groups on the impact of the events described in the “ghost fishing net” scenario than for other scenarios. Further comparison of the unalike scores between participants with penguin biology or conservation experience (*N* = 18) and those participants with penguin experience in other areas (e.g., ecotourism, management, *N* = 24) again indicated poor agreement within these groups with unalike scores between 0.31 and 0.83 (Supplementary Table 2).

## Discussion

In summary, our bibliometric analysis of the literature on the anthropogenic factors faced by wild penguins indicated five main themes, broadly described as human disturbance through tourism, climate change, pollution, fisheries and the spread of emerging diseases. Participants scored the overall impact on penguins as negative with mean scores for mental state above 5/10 for all scenarios. The effect of being trapped in a ghost fishing net was rated as having a significantly more negative effect than events described in the other scenarios. Scores provided by participants for each domain and the duration of negative effect for each scenario were largely as expected. We found that participants with different work or scientific interests and different levels of experience of working with penguins scored similarly on all domains and scenarios. Although the above findings indicate reasonable assessments of the impact of the scenarios on each domain, there was disagreement between participants. Contrary to our hypothesis, participants with experience of working with penguins, or those that had worked with penguins for more time, showed no better agreement in the scores given compared to other participants. Our findings indicate that anthropogenic factors are generally considered to severely impact penguin welfare, but within different groups of participants there is considerable disagreement on the extent of this negative impact.

Our bibliometric analysis grouped the anthropogenic factors that impact penguins into five main themes, and on average participants considered these to be considerable threats to wild penguin welfare. Our analysis supports the findings of Trathan et al. (27) who regarded that pollution, habitat degradation and loss and fishing were the most important threats to penguins. In our study, participants perceived being trapped in a ghost fishing net as having the most severe negative effect on welfare, and research on this issue confirms that being trapped in a net causes significant bruising and internal hemorrhaging (36). The high rating for the ghost fishing net scenario by our participants suggest that they may have been particularly attuned to high-intensity, short-term impacts on welfare. Conversely, more longer-term and lower-intensity impacts such as those due to climate change or mercury accumulation were scored as having the least impact on welfare. The above findings raise the possiblity that interest groups may regard welfare as more compromised by short, high-intensity events. Additionally, as we continue to explore perceptions of animal welfare, it would be important to determine the influence of words and descriptions on evaluations. Although we took care to write the scenarios to provide only objective indicators of biological function, it is possible that some of our word choices and descriptions may have biased the participants' ratings. As we advance the use of the five domains model to assess welfare in an increasing number of different contexts, it would be useful to understand how, or if, word choice and descriptions influence welfare assessment.

There are several indications that the interest groups' assessment of welfare was supported by the PWAT. First, the domain with the highest score for each scenario was mostly as expected. For example, “environment” was scored highest for the ice melt scenario and “health” scored the highest for the IBDV infection scenario. Second, one concern with successively rating different events was that participants may shift in their assessment, whether to a more positive or a more negative rating as they progressively assess each scenario. However, our examination of the effect of the order of presentation of each scenario on the scores given for each domain did not provide any evidence that on the whole participants shifted in their assessments as they progressed through the five scenarios. Third, we found good correlation between the physical domains and “mental state,” which suggests that the tool was facilitating a coherent assessment of welfare. It was interesting to note that the score for the mental state was lower than at least one physical domain score for three of the five scenarios. Our findings suggest that participants considered that some physical impacts were not exerting an effect on mental state, for example such as if the penguin had no sensation or symptoms of a particular physical condition. On purpose, we refrained from providing too much guidance and instructions as to how the PWAT should be used, and even changed the usual “affect” label to “mental state” as we considered this would be easier to understand. We believe these changes are within the remit of the FDM as a flexible facilitatory device that should not be adhered to rigidly and dogmatically (23).

One advantage of examining the issue of penguin welfare within a citizen science framework was that it allowed us to examine the human dimension in welfare assessment. Perception of the welfare of farms animals has been considered to be constructed based on people's frames of reference (37). Frames of reference are constructed by convictions (opinions about “the way things are”), values (opinions about “the way things should be”), norms (rules of conduct) and knowledge and interests (including economic, social and moral interests) (38, 39). We found no evidence that participants that worked or studied penguins, and hence may be expected to have greater knowledge or interest of penguin biology scored differently to other people. People belonging to animal welfare or conservation organizations may also have had different convictions and values to those not belonging to such organizations, but again no evidence was found that these groups of participants assessed welfare differently. Our findings are in line with research on the role of values in the rhino horn trade debate, where people with biospheric values (i.e., people concerned with problems affecting all living things) do not show more support for banning this trade compared to other people (40). We propose that continuing to understand the perceptions of animal welfare by interest groups is essential to identify wider ethical concerns, guide dialogue and prioritize remedial action (41).

Surprisingly, we found that the level of agreement among participants with considerable years of experience of working or studying penguins was not better than among participants without experience of working with penguins. When this was examined further, participants in the “penguin biology and conservation” group were no more likely to agree than those working with penguins in other areas. As outlined in the introduction, assessing animal welfare is complex and previous attempts to assess the welfare of wild animals have relied on assessors with expert knowledge of animal welfare (8, 16). Although our findings suggest that experience of penguins *per se* was not a major cause of the levels of disagreement among assessors, perhaps more likely is the quality and availability of the background data.

It is reasonable to expect that the more background data on the biology and welfare of the species is provided, then the greater the accuracy and agreement on the welfare state of the individuals. For example, Nicol et al. (16) provided a 3,000-word background factsheet on the relevant science to inform assessors, and reported good agreement between expert assessors. This approach was not possible in our study because we wanted to examine a wide range of scenarios with a large and diverse group of participants, and it would have been excessively time-consuming to ask participants to familiarize themselves with comprehensive background information on penguin biology and welfare. Additionally, little is known about the impact of some anthropogenic factors on penguin biology and welfare, making the development of factsheets difficult. High assessor agreement has previously been considered as an important factor in the success of tools to assess animal welfare (14, 23), and research is required to obtain objective indicators of the impact of anthropogenic factors on penguin biology to assist welfare assessment.

In conclusion, participants with an interest in penguins, seabird biology or wildlife conservation generally perceived that typical anthropogenic environmental factors have a negative impact on penguin welfare, including on physical aspects (nutrition, environmental, health, and behavior) as well as their mental state. Short-term and high-intensity events were regarded as having a more severe impact on welfare, indicating that interest groups may be more sensitive to these impacts than to longer-term, less intense events. However, our analyses of agreement within groups underlined the need for scientific knowledge of penguin biological responses to anthropogenic factors to facilitate the accurate assessment of wild penguin welfare. Our findings highlight the importance of scientific knowledge of penguin biological responses to anthropogenic factors to support progress in the assessment of penguin welfare.

## Data Availability Statement

The raw data supporting the conclusions of this article will be made available by the authors, without undue reservation.

## Ethics Statement

The studies involving human participants were reviewed and approved by Charles Sturt University Ethics in Human Research Committee. The participants provided their written informed consent to participate in this study.

## Author Contributions

All authors listed have made a substantial, direct and intellectual contribution to the work, and approved it for publication.

## Conflict of Interest

The authors declare that the research was conducted in the absence of any commercial or financial relationships that could be construed as a potential conflict of interest.

## Publisher's Note

All claims expressed in this article are solely those of the authors and do not necessarily represent those of their affiliated organizations, or those of the publisher, the editors and the reviewers. Any product that may be evaluated in this article, or claim that may be made by its manufacturer, is not guaranteed or endorsed by the publisher.

## References

[1] Ohl F Van der Staay FJ. Animal welfare: at the interface between science and society. Vet J. (2012) 192:13–9. 10.1016/j.tvjl.2011.05.019 21703888

[2] Feber RE Raebel EM D'cruze N Macdonald DW Baker SE. Some animals are more equal than others: wild animal welfare in the media. Bioscience. (2017) 67:62–72. 10.1093/biosci/biw144

[3] Ramp D Bekoff M. Compassion as a practical and evolved ethic for conservation. Bioscience. (2015) 65:323–7. 10.1093/biosci/biu223

[4] Dubois S Fenwick N Ryan EA Baker L Baker SE Beausoleil NJ. International consensus principles for ethical wildlife control. Conserv Biol. (2017) 31:753–60. 10.1111/cobi.12896 28092422

[5] Hampton JO Hyndman TH. Underaddressed animal-welfare issues in conservation. Conserv Biol. (2019) 33:803–11. 10.1111/cobi.13267 30549308

[6] Dubois S Fraser D. Rating harms to wildlife: a survey showing convergence between conservation and animal welfare views. Anim Welfare. (2013) 22:49–55. 10.7120/09627286.22.1.049

[7] Wallach AD Bekoff M Batavia C Nelson MP Ramp D. Summoning compassion to address the challenges of conservation. Conserv Biol. (2018) 32:1255–65. 10.1111/cobi.13126 29700860

[8] Beausoleil NJ Mellor DJ Baker L Baker SE Bellio M Clarke AS. “feelings and fitness” not “feelings or fitness”–the raison d'être of conservation welfare, which aligns conservation and animal welfare objectives. Front Vet Sci. (2018) 27:296. 10.3389/fvets.2018.00296 30538995 PMC6277474

[9] Hampton JO Warburton B Sandøe P. Compassionate versus consequentialist conservation. Conserv Biol. (2019) 33:751–9. 10.1111/cobi.13249 30411399

[10] Boogaard BK Oosting SJ Bock BB. Elements of societal perception of farm animal welfare: a quantitative study in The Netherlands. Livest Sc. (2006) 104:13–22. 10.1016/j.livsci.2006.02.010

[11] McIntosh D Wright PA. Emotional processing as an important part of the wildlife viewing experience. J Outdoor Recreat and Tour. (2017) 18:1–9. 10.1016/j.jort.2017.01.004

[12] Coleman G. Public animal welfare discussions and outlooks in Australia. Anim Front. (2018) 8:14–9. 10.1093/af/vfx004 32002210 PMC6952000

[13] De la Fuente MF Souto A Caselli C Schiel N. People's perception on animal welfare: why does it matter? Ethnobiol Conserve. (2017) 6:7. 10.15451/ec2017-10-6.18-1-7

[14] Baker SE Sharp TM Macdonald DW. Assessing animal welfare impacts in the management of European rabbits (*Oryctolagus cuniculus*), European moles (*Talpa europaea*) and Carrion crows (*Corvus corone*). PLoS ONE. (2016) 11:e0146298. 10.1371/journal.pone.0146298 26726808 PMC4699632

[15] Beausoleil NJ Fisher P Littin KE Warburton B Mellor DJ Dalefield RR. systematic approach to evaluating and ranking the relative animal welfare impacts of wildlife control methods: poisons used for lethal control of brushtail possums (*Trichosurus vulpecula*) in New Zealand. Wildlife Res. (2016) 43:553–65. 10.1071/WR16041

[16] Nicol C Bejder L Green L Johnson C Keeling L Noren D. Anthropogenic threats to wild cetacean welfare and a tool to inform policy in this area. Front Vet Sci. (2020) 7:57. 10.3389/fvets.2020.00057 32185183 PMC7058697

[17] Broom DM. Indicators of poor welfare. Brit vet J. (1986) 142:524–6. 10.1016/0007-1935(86)90109-0 3594185

[18] Bracke MBM De Greef KH Hopster H. Qualitative stakeholder analysis for the development of sustainable monitoring systems for farm animal welfare. J Agric Environ Ethic. (2005) 18:27–56. 10.1007/s10806-004-3085-2

[19] Irwin A. Citizen Science: A Study of People, Expertise and Sustainable Development. New York, NY: Routledge (1995). p. 202.

[20] Crain R Cooper C Dickinson JL. Citizen science: a tool for integrating studies of human and natural systems. Annu Rev Env Resour. (2014) 39:641–65. 10.1146/annurev-environ-030713-154609

[21] McKinley DC Miller-Rushing AJ Ballard HL Bonney R Brown H Cook-Patton SC. Citizen science can improve conservation science, natural resource management, and environmental protection. Biol Conserv. (2017) 208:15–28. 10.1016/j.biocon.2016.05.015

[22] Mellor DJ Reid CSW. Concepts of animal well-being and predicting the impact of procedures on experimental animals. In: Baker RM Jenkins G Mello DJ, editors. Improving the Well-being of Animals in the Research Environment. Glen Osmond, SA: Australian and New Zealand Council for the Care of Animals in Research and Teaching (1994). p. 3–18.

[23] Mellor DJ. Operational details of the five domains model and its key applications to the assessment and management of animal welfare. Animals. (2017) 7:60. 10.3390/ani7080060 28792485 PMC5575572

[24] Mittermeier JC Correia R Grenyer R Toivonen T Roll U. Using Wikipedia to measure public interest in biodiversity and conservation. Conserv Biol. (2021) 2021:13702. 10.1111/cobi.13702 33749051

[25] Boersma PD. Penguins as marine sentinels. Bioscience. (2008) 58:597–607. 10.1641/B580707

[26] International Union for Conservation of Nature. The IUCN Red List of Threatened Species. (2021). Available online at: https://www.iucnredlist.org (accessed April 20, 2021).

[27] Trathan PN García-Borboroglu P Boersma D Bost CA Crawford RJ Crossin GT. Pollution, habitat loss, fishing, and climate change as critical threats to penguins. Conserv Biol. (2015) 29:31–41. 10.1111/cobi.12349 25102756

[28] Harvey AM Beausoleil NJ Ramp D Mellor DJ. A ten-stage protocol for assessing the welfare of individual non-captive wild animals: free-roaming horses (*Equus ferus caballus*) as an example. Animals. (2020) 10:148. 10.3390/ani10010148 31963232 PMC7022444

[29] Van Eck NJ Waltman L. Visualizing bibliometric networks. In: Ding Y Rousseau R Wolfram D, editors. Measuring Scholarly Impact. Cham: Springer (2014). p. 285–320. 10.1007/978-3-319-10377-8_13

[30] de Vet HC Terwee CB Knol DL Bouter LM. When to use agreement versus reliability measures. J Clin Epidemiol. (2006) 59:1033–9. 10.1016/j.jclinepi.2005.10.015 16980142

[31] Kader GD Perry M. Variability for categorical variables. J Stat Edu. (2017) 1:15. 10.1080/10691898.2007.11889465

[32] Fox N Ward K. Health, ethics and environment: a qualitative study of vegetarian motivations. Appetite. (2008) 50:422–9. 10.1016/j.appet.2007.09.007 17980457

[33] RStudio Team. RStudio: Integrated Development for R. Boston, MA: RStudio, PBC. (2020). Available online at: http://www.rstudio.com/ (accessed May 10, 2021).

[34] Bauer DJ Sterba SK. Fitting multilevel models with ordinal outcomes: performance of alternative specifications and methods of estimation. Psychol Methods. (2011) 16:373. 10.1037/a0025813 22040372 PMC3252624

[35] Burnham KP Anderson DR. A Practical Information-Theoretic Approach. 2nd ed. New York, NY: Springer-Verlag (2002). p. 488. 10.1007/b97636

[36] Darby JT Dawson SM. Bycatch of yellow-eyed penguins (*Megadyptes antipodes*) in gillnets in New Zealand waters 1979–1997. Biol Conserv. (2000) 93:327–32. 10.1016/S0006-3207(99)00148-2

[37] Vanhonacker F Verbeke W Van Poucke E Tuyttens FA. Do citizens and farmers interpret the concept of farm animal welfare differently? Livest Sci. (2008) 116:126–36. 10.1016/j.livsci.2007.09.017

[38] Kickert WJ Klijn EH Koppenjan JF. Introduction: a management perspective on policy networks. In: Kickert WJ Klijn EH Koppenjan JF, editors, Managing Complex Networks: Strategies for the Public Sector. London: Sage (1997). p. 1–11. 10.4135/9781446217658.n1

[39] Te Velde H Aarts N Van Woerkum C. Dealing with ambivalence: farmers' and consumers' perceptions of animal welfare in livestock breeding. J Agr Environ Ethic. (2002) 15:203–19. 10.1023/A:1015012403331

[40] Brown AA Dean AJ Possingham H Biggs D. The role of animal welfare values in the rhino horn trade debate. Conser Sci Pract. (2019) 11:e103. 10.1111/csp2.104

[41] Toomey AH Domroese MC. Can citizen science lead to positive conservation attitudes and behaviors? Hum Ecol Rev. (2013) 1:50–62. Available online at: https://www.jstor.org/stable/24707571

