# Supplementary material for: A Citizen Science Trial to Assess Perception of Wild Penguin Welfare
Source: Front Vet Sci. 2021 Jul 27;8:698685. doi: 10.3389/fvets.2021.698685 (PMC8353176; doi:10.3389/fvets.2021.698685)

Supplementary material

Identification of relevant themes and development of the scenarios

The literature search yielded 2801 items; of which 347 were classified as “possible anthropogenic effects”, 1971 items were on penguins but not to do with anthropogenic effects, and 493 were not on penguins (e.g. there is a “penguin effect” in physics). A co-occurrence map of the 347 items identified as possible anthropogenic effects was created using VOS viewer. The unit of analysis was all keywords and a full counting method was used. By default, VOSviewer identified 176 words that met the threshold of occurring at least 5 times and grouped these into five clusters (Figure S1). The main themes and words in each of these clusters is presented in Table 1 and were used to develop five hypothetical scenarios (Box 1). Each scenario provided information on the biology of an individual penguin and care was taken to avoid leading comments on the welfare state of the individual.

Table S1: Table showing the number of participants from different areas of expertise and their experience of working or studying penguins. (64 participants answered these questions).

		**Experience working or studying with penguins**				
		None	Less than 5 years	6-10 years	More than 10 years	**Total**
**Expertise**	Penguin biology or conservation	0	4	10	4	**18**
	Other biology or conservation	9	7	2	6	**24**
	Ecology	4	1	2	2	**9**
	Wild animal management	1	0	2	0	**3**
	Other	8	1	1	0	**10**
	**Total**	**22**	**13**	**17**	**12**	

Table S2: Unlike scores based on the 5-point-scale for each domain and scenario for participants that work in penguin biology or conservation (n=18) and participants that work with penguins in other areas (n=24).

Domain	Scenario	Penguin biology or conservation (n=18)	Penguin experience in other areas (n=24)
Nutrition	Tourism	0.71	0.75
	Ice melt	0.76	0.76
	Mercury accumulation	0.72	0.76
	Ghost net	0.79	0.75
	IBDV infection	0.79	0.74
Environment	Tourism	0.77	0.81
	Ice melt	0.66	0.75
	Mercury accumulation	0.76	0.76
	Ghost net	0.31	0.58
	IBDV infection	0.83	0.77
Health	Tourism	0.75	0.77
	Ice melt	0.61	0.78
	Mercury accumulation	0.71	0.76
	Ghost net	0.45	0.53
	IBDV infection	0.60	0.71
Behavior	Tourism	0.76	0.79
	Ice melt	0.67	0.77
	Mercury accumulation	0.71	0.81
	Ghost net	0.65	0.63
	IBDV infection	0.65	0.79
Mental State	Tourism	0.75	0.82
	Ice melt	0.73	0.81
	Mercury accumulation	0.74	0.82
	Ghost net	0.39	0.53
	IBDV infection	0.78	0.79

Figure S1: VOSviewer visualisation of a network of all keywords in 347 articles from 2010-2020 on anthropogenic effects on penguins. Size of nodes indicated the number of times keywords appeared and nearness of nodes and lines indicates terms that co-occur. Colours indicated the clusters generated by VOSviewer.

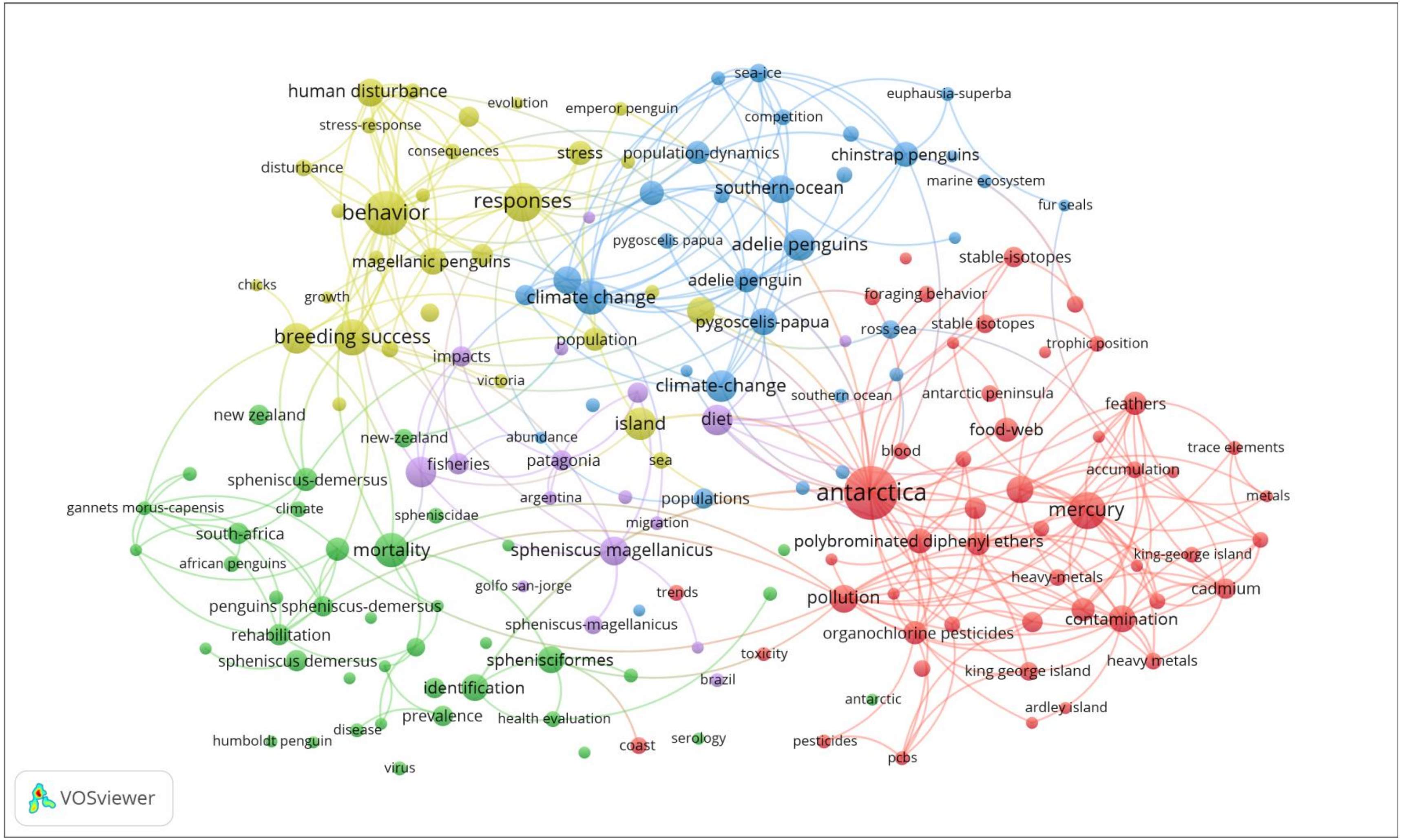

Supplement: Supplementary file 1 [file Data_Sheet_1.pdf]
